# Isolation of Infectious Bursal Disease Virus Using Indigenous Chicken Embryos in Kenya

**DOI:** 10.1155/2015/464376

**Published:** 2015-11-23

**Authors:** W. U. Mutinda, L. W. Njagi, P. N. Nyaga, L. C. Bebora, P. G. Mbuthia, D. Kemboi, J. W. K. Githinji, A. Muriuki

**Affiliations:** ^1^State Department of Livestock, Regional Veterinary Investigation Laboratories, P.O. Box 204-80113, Mariakani, Kenya; ^2^University of Nairobi, P.O. Box 29053-00625, Nairobi, Kenya; ^3^State Department of Livestock, Central Veterinary Laboratories, Private Bag Box 00625, Nairobi, Kenya; ^4^Kenya Veterinary Vaccine Production Institute (KEVEVAPI), P.O. Box 53260-00200, Nairobi, Kenya

## Abstract

Infectious bursal disease virus (IBDV) isolates were recovered from outbreaks to initiate activities towards developing a local vaccine strain. Use of indigenous chicken embryos was exploited to determine their potential, promote utilization of local resources for research, and enhance household economic activities. Bursa of Fabricius (BFs) samples from outbreaks shown to be IBDV positive was homogenized and inoculated in 4-week-old specific pathogen-free (SPF) IBDV seronegative white leghorn chicks. The harvested virus was inoculated into 11-day-old indigenous chicken embryos that were IBDV seronegative and passaged serially three times after which they were inoculated into 4-week-old indigenous chicks to test for presence and virulence of propagated virus. Out of 153 BFs collected from outbreaks, 43.8% (67/153) were positive for IBDV antigen and 65.7% (44/67) caused disease in SPF chicks. The embryo mean mortalities were 88% on primary inoculation, 94% in 1st passage, 91% in 2nd passage, and 67% in 3rd passage. After the third passage in embryos all the 44 isolates were virulent in 4-week-old indigenous chicks. The results show that indigenous chicken embryos support growth of IBDV and can be used to propagate the virus as an alternative viral propagating tool for respective vaccine preparation.

## 1. Introduction

Infectious bursal disease (IBD), a highly contagious immunosuppressive disease of young chickens [1], is caused by infectious bursal disease virus (IBDV). The virus is a member of the family Birnaviridae and the genus* Avibirnavirus*. There are two known serotypes of IBDV. Serotype 1 is pathogenic to chickens in which it causes IBD, while serotype 2 is apathogenic. The two serotypes neither cross-protect nor cross-neutralise each other [1].

Chickens infected with IBDV between 3 and 6 weeks of age develop clinical IBD which may result in death but those infected at less than 3 weeks of age usually have few or no clinical signs. The disease has also been observed in chickens older than 6 weeks, even in up to 20-week-old chickens [2, 3]. Irrespective of when the infection occurs the disease causes immune-suppression which makes the birds vulnerable to a variety of secondary infections. As a result, infected chickens develop a poor immune response to vaccination against other pathogens [4–6]. Infectious bursal disease is one of the most economically important diseases that affects commercially produced chickens worldwide [1, 7, 8]. In Kenya, the disease is of economic importance in exotic as well as free range indigenous chickens [3].

Imported vaccines are used to control the disease in Kenya [9]. Vaccination failure has been a big challenge in the control of this disease. The IBD viruses like many RNA viruses evolve quickly because of the low proofreading activity of their viral replicase [10]. Due to this inherent property and other additional evolutionary selection processes in the field high variability of the viral genome is observed [10]. Adamu et al. [11], while investigating the phylogenetic relationship between field and foreign vaccine strains in Nigeria, found that when IBDV strains spread from their region of origin to a different region they mutate alongside indigenous field strains. Antigenic variation is, therefore, a possible cause of vaccination failure [11]. Farmers in Kenya experience outbreaks of this disease in vaccinated flocks [9]. This could be due to a mismatch between the local strains that cause the disease and immune response induced by imported vaccines. A vaccine designed out of the local strains would provide good protection against the same local strains.

Isolation of IBDV is usually carried out using specific antibody-negative (SAN) chickens, cell cultures, or specific pathogen-free (SPF) embryonating eggs from specific antibody-negative sources [12]. However, some difficulty may be experienced in using the latter two systems as the virus does not readily adapt to them and sometimes availability of SPF hybrid embryos may be a challenge. Strains of IBDV tend to show reduced virulence when passaged in embryonated eggs [13, 14]. A study done in Bangladesh to compare isolation of IBDV using commercial hybrid chicken embryos and indigenous chicken embryos [15] showed that rural indigenous chicken eggs were better for virus isolation than commercial farm hybrid chicken eggs. Strains of IBDV circulating in North Africa [16, 17] and West Africa [18] have been isolated for characterization and vaccine development but not in East Africa. Currently, there is no report available on isolation of local IBDV strains using embryonated indigenous chicken eggs in Kenya. This study fills this gap.

## 2. Materials And Methods

### 2.1. Study Design

Bursa of Fabricius (BFs) samples collected from birds and fresh carcasses from infectious bursal disease (IBD) outbreaks was used as sources of respective virus samples and those from the same farm were pooled and homogenized together. Presence of the viral antigen in the BFs was confirmed using agar gel precipitation test (AGPT). Positive bursa samples were used to inoculate 4-week-old SPF IBDV seronegative white leghorn chicks. Birds were preferred for initial sample inoculation meant to amplify the virus from field samples, since other methods could modify the original characteristics of the IBDV field strains [19]. White leghorn chicks have been shown to have the highest IBDV antigen load in the bursal tissues compared to other IBDV infected chicks [20]. After 72 hours, respective BFs were aseptically harvested into universal bottles and stored at −20°C. For convenient referencing, the set of bursae from outbreak cases was referred to as “first-generation bursae” while bursae and viruses harvested from SPF white leghorns were referred to as “second-generation bursae and viruses, respectively.” The second-generation bursae were used as sources of virus for propagation through indigenous chicken embryos. The viruses were serially passaged three times in SAN indigenous chicken embryos, after which the embryo and chorioallantoic membrane (CAM) combined homogenate harvested from the 3rd passage were inoculated into 4-week-old IBDV seronegative indigenous chicks, which served as indicators for viral presence and virulence. Presence of virus in the bursae of these birds was confirmed by AGPT using known antiserum. The bursae and viruses obtained from inoculated indigenous chickens were referred to as “third-generation bursae and viruses, respectively.”

### 2.2. Animal Welfare

Permission to use chickens in the experiments was granted by the Biosecurity, Animal use and Ethics Committee of the Faculty of Veterinary Medicine, University of Nairobi. The birds were handled according to the internationally accepted regulations and ethical considerations in animal experiments [21].

### 2.3. Experimental Birds

Both white leghorn and indigenous chicks were hatched and raised to the age of 4 weeks at the University of Nairobi, Kabete Campus. The white leghorn chicks were hatched from embryonated eggs obtained from a specific pathogen-free (SPF) flock maintained at Kenya Veterinary Vaccine Production Unit (KEVEVAPI) of the Government of Kenya. Indigenous chicks were hatched from fertile eggs obtained from indigenous chickens that were kept in an isolated farm with no history of IBD outbreak and were maintained unvaccinated against IBDV. They were mainly normal feathered birds with a few naked neck types. Sera from these indigenous chickens were confirmed to be free from IBDV antibodies and Newcastle disease virus antibodies through AGPT [12] and haemagglutination inhibition test [22], respectively.

### 2.4. Experimental Embryos

Indigenous chicken embryos were utilized at 11 days old for the virus propagation and serial passage experiment. They were obtained from the same farm of SAN indigenous chickens described above that supplied the eggs which hatched to indigenous chicks. Strict biosecurity measures were observed all the time. The embryos were tested for IBD antibodies before use and were found negative.

### 2.5. Source of First-Generation Bursa of Fabricius Samples

Bursa of Fabricius (BF) samples was aseptically collected from suspected infectious bursal disease outbreaks in layers, broilers, and indigenous chickens in Kenya. Collection centres where postmortem examinations were done were National Central Veterinary Laboratories, Kabete; Regional Veterinary Investigation laboratories (RVIL) at Mariakani, Ukunda, and Nakuru, University of Nairobi Poultry Clinic; and Nakuru Veterinary Resource Center. The samples were submitted to University of Nairobi Virology Laboratory under cold chain.

### 2.6. Processing of Bursa of Fabricius Samples

Bursa of Fabricius samples was homogenized into 50% (w/v) suspension in phosphate buffered saline (PBS). The homogenate was centrifuged at 2000 rpm for 30 minutes and the supernatant was harvested and tested for viral antigen by the AGPT [12, 23]. Positive samples were treated with penicillin (1000 units) and streptomycin at 1000 *μ*g/mL per sample [12] and kept at −20°C in 2 mL aliquots until used.

### 2.7. Inoculation of White Leghorn Chicks

Four-week-old IBD antibody-negative white leghorn chicks were inoculated with 100 *μ*L of the antibiotic-treated AGPT-positive, first-generation, bursal samples via intranasal and eye-drop routes. A fifty-microlitre volume was given intranasally and another fifty-microlitre volume was given by intraocular route [24]. Three birds were used for each sample. The chicks were killed 72 hours after inoculation, and the carcasses were examined for lesions, and the BFs were harvested aseptically. Nonhaemorrhagic BFs harvested from birds inoculated with same sample were pooled and homogenized together. Presence of second-generation virus was confirmed by AGPT test. The AGPT-positive samples were treated with antibiotics, as previously described [12], and stored at −20°C for propagation through IBDV seronegative indigenous chicken embryos.

### 2.8. Inoculation of Indigenous Chicken Embryos

Each AGPT-positive second-generation bursal suspension was inoculated into the CAM of three 11-day-old SAN indigenous chicken embryos at a dose of 200 *μ*L of viral suspension per embryo [12]. The inoculated embryonated eggs were incubated at 37°C for six days and candled twice daily. The embryos that died within 48 hours of incubation were discarded while those that died after 48 hours were recorded and chilled at 4°C. The experiment was terminated on day six after inoculation and the remaining live embryonated eggs were chilled at 4°C overnight. Three serial embryo passages were performed. At each passage, embryos together with the CAMs were harvested aseptically into a petri dish and examined and observed lesions were recorded. The embryo head and limbs were discarded. The main body of the embryo was homogenized together with the CAM in PBS to make an embryo suspension [12]. Harvested supernatant was treated with penicillin (1000 units) and streptomycin (1000 *μ*g/mL) per sample [12] and passaged by reinoculating into fresh embryos and repeating the process three times. At the end of every passage AGPT was done to check for presence of IBDV in the embryo CAM combined homogenate.

### 2.9. Inoculation of Indigenous Chicks

Harvested virus homogenate from the 3rd embryo passage was inoculated into 4-week-old IBDV antibody-negative indigenous chicks. Each chick was inoculated with 1 mL of harvested homogenate via the oral and oculonasal routes [24]. Three birds were inoculated per sample. Birds were observed for clinical signs of IBD and BFs were harvested aseptically 72 hrs after inoculation from each inoculated bird. Presence of IBDV in the bursa was confirmed by AGPT and labeled as third-generation bursa and virus, respectively.

### 2.10. Agar Gel Precipitation Test

Materials from respective bursae were prepared and AGPT was done as previously described [3, 12]. Standardized antigen, Cat number RAA0123 (IBDV antigen), and standardized antisera, Cat number RAB0124 (IBDV type 1 +ve serum), used were imported from Animal Health and Veterinary Laboratories Agency, United Kingdom.

## 3. Results

### 3.1. Clinical Field Cases Antigen Detection

Out of one hundred and fifty-three (153) BFs submitted from suspected IBD outbreaks, 67 (43.8%) were positive for IBDV on AGPT test (Table 1). When the 67 IBDV positive samples were inoculated into white leghorn chicks 44 (65.7%; 44/67) infected the chicks and caused disease; viral antigen was detected in all the 44 harvested B/F samples by AGPT.

### 3.2. Clinical Signs and Lesions in White Leghorn Chicks

Clinical signs observed in white leghorn chicks after inoculation were inappetence, ruffled feathers, white watery diarrhea, depression, and death. On opening the carcasses, typical IBD lesions were observed; they included enlarged BFs which were also oedematous, haemorrhagic, and sometimes necrotic and atrophied, with caseous cheesy exudates in the mucosa, haemorrhages in the thigh, leg, and breast muscles, proventriculus, caecal tonsils, thymus, and spleen. In addition, the spleen, caecal tonsils, and thymus were swollen.

### 3.3. Lesions on Inoculated Indigenous Chicken Embryos

**Figure 1:**
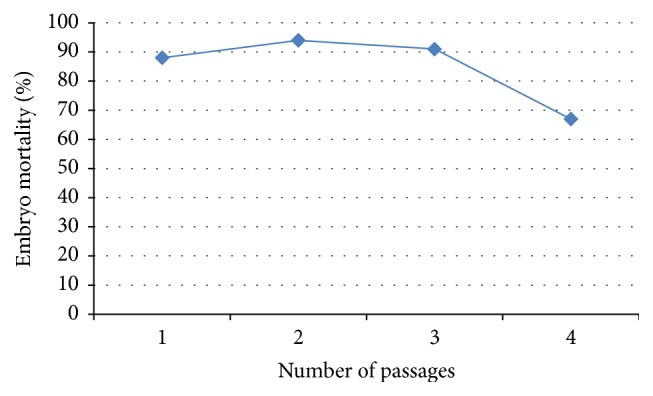
Percent mortality in different passages of embryos inoculated with infectious bursal disease virus.

**Figure 2:**
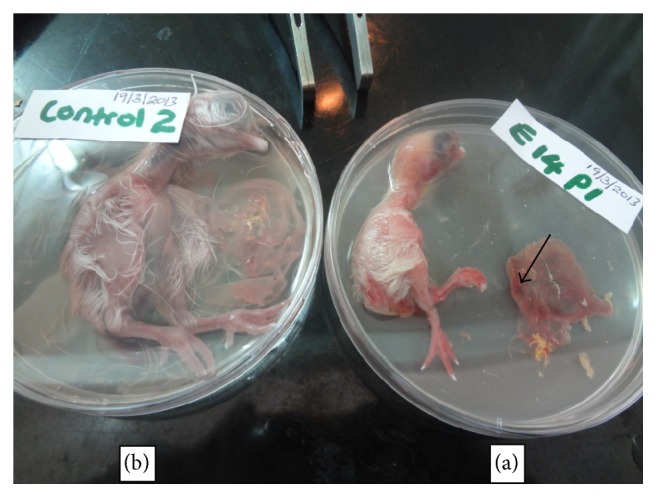
Eighteen-day-old dwarf congested IBDV infected embryo (a) with haemorrhagic chorioallantoic membrane (black arrow in (a)) as compared to the uninfected 18-day-old control (b).

**Figure 3:**
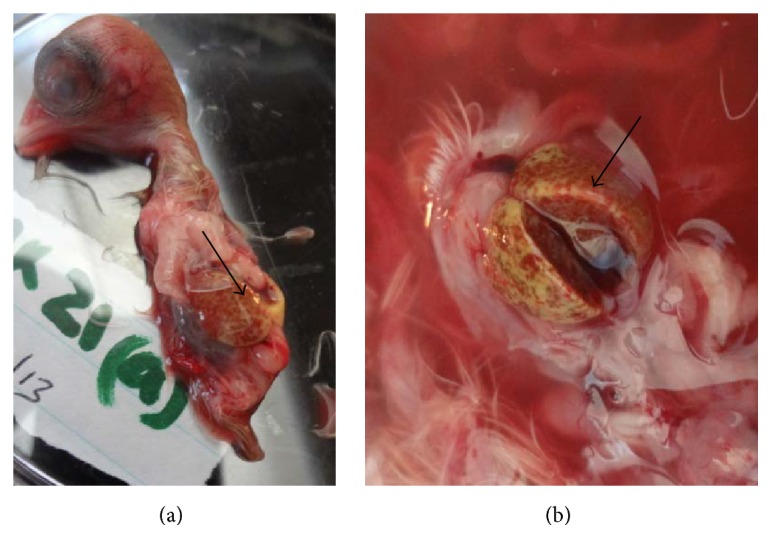
Swollen liver with patchy congestion and pale yellow-green colouration producing a mottled effect (black arrows in (a and b); (b) a closer view of the liver) in an indigenous chicken embryo inoculated with infectious bursal disease virus.

A summary of the lesions observed when the isolates were passaged in 11-day-old indigenous chicken embryos is shown in Table 2. All isolates were similar in growth pattern and effect on embryos. The most common observation was death of the embryos. The embryo mortality rate was 88% on primary inoculation; it then rose to 94% in 1st passage and 91% in 2nd passage and then came down to 67% in 3rd passage (Figure 1). Embryo mortality was high between day 3 and 4 after inoculation. Dwarfed embryos (Figure 2) with oedema and congestion followed as the next common lesion. The livers were swollen with patchy congestion (Figure 3) and pale yellow colour (in some parts tending to green) showing mottling. Kidneys and spleen were also enlarged with patchy congestion observed in primary inoculation. Oedematous chorioallantoic membranes (CAMs) were observed with congestion or haemorrhages, as shown in Table 2 and Figure 2. In general there was an overall reduction of lesions observed in the embryos with increased passage as shown in Table 2. Agar gel precipitation test done to confirm the presence of the virus in the embryos yielded faint precipitation lines which, though hardly visible, were present in all the passages.

### 3.4. Lesions Observed on Inoculated Indigenous Chicks

**Figure 4:**
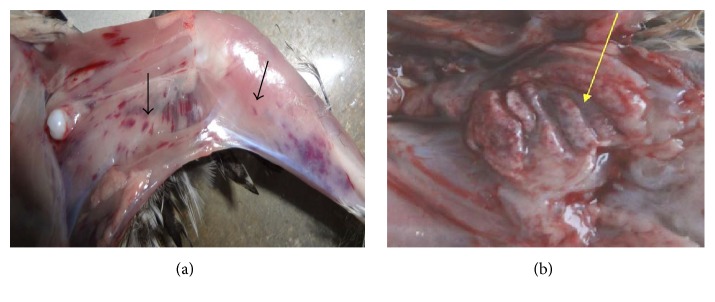
Showing haemorrhages on the thigh and leg muscles (arrows in picture (a)) and in an opened bursa (see arrow in (b)) following inoculation of indigenous chicken with 3rd egg passage virus.

When inoculated into indigenous chicks (as indicators), all the 44 embryo-passaged viral isolates (after the third passage) produced disease. The main clinical signs observed were watery diarrhoea, ruffled feathers, reluctance to move, anorexia, trembling, and prostration. Postmortem lesions included dehydration of the skeletal muscles with numerous ecchymotic and petechial haemorrhages (Figure 4) and enlargement of the kidneys with urate-distended tubules. The bursa of Fabricius showed lesions characteristic of IBD (Figure 4) and was enlarged and turgid. Caseous necrotic debris was observed in the lumen of the bursa. Intrafollicular haemorrhages were present and, in some cases, the bursa was completely haemorrhagic giving the appearance of a black cherry. Peribursal straw-coloured oedema was present in many bursae. All the BFs harvested from the inoculated indigenous chicks had viral antigen confirmed by the AGPT; they yielded clear distinctly visible precipitation lines against the reference antiserum.

## 4. Discussion

The purpose of cultivating IBDV in chicken embryos was to determine whether the virus could be adapted through passaging in the indigenous chicken embryos. The indigenous chickens used in the experiments as well as the source of the indigenous chicken embryos were not inbred animals. Indigenous chickens in the households of Kenya consist of a nonselected heterogeneous population that is evenly distributed across the country [25]. As in most other developing countries in the tropics the chickens have not yet been classified into breeds [25]. They are commonly named according to regions of placements or ecotypes or phenotypic expression of major genes [26]. In this study the birds used were mainly a population of normal feathered birds with a few naked neck types. The chicken population in most counties in Kenya is dominated by normal feathered genotype [27]. Previous research found that indigenous chickens were genetically related for Kenya, Uganda, Ethiopia, and Sudan birds but were distinct from commercial exotic broiler and layer lines [28].

The results of this study compare well with other studies in which indigenous chicken embryos were reported to be good for virus isolation [15]. In this study all the 44 isolates recovered from the white leghorn chicks grew in indigenous chicken embryos. Snedeker et al. [29] and Izawa et al. [30] made serial passages of the virus in chicken embryos resulting in adaptation of the virus and attenuation of very virulent and classic strains. Furthermore, Ahmad et al. [31] reported reduction in mortality as virus was passaged in embryonated eggs until very low mortalities were seen in the sixth passage. Lesions observed in indigenous chicken embryos inoculated with the Kenyan IBDV isolates were as reported for IBDV strains inoculated in embryos of other types of chicken [12, 24]. High mortality of inoculated embryos, oedema, congestion, and haemorrhages observed in embryos in this study have in the past been associated with hypervirulent and classic strains [32, 33]. Dwarfing of embryos and enlarged liver and spleen observed in this study were comparable to observations reported by Lukert and Saif [34]. Very weak precipitation lines which were hardly visible when AGPT was used to test for the presence of viral antigen in the harvested embryos were an indication that virus quantity was low in the embryos. This finding agrees with findings by other researchers that IBDV strains do not grow easily in embryonated chicken eggs and take time to adapt [14]. However, when, in this study, the embryo homogenates from the last passage were inoculated into indigenous chicks, the disease was reproduced by all the isolates. Agar gel precipitation test done to detect viral antigen in the bursae of these chicks yielded highly visible and very strong precipitation lines. The isolates were still virulent after 3 serial passages in indigenous chicken embryos. Variations in the embryo passage numbers leading to IBDV attenuation have been observed by researchers and attributed to experimental conditions, strain of the virus, and internal environment in the eggs [14]. Attenuation of IBDV in embryos was obtained after 43 passages by Lazarus et al. [35], after 8 serial passages by Yamaguchi et al. [36], and after 13 serial passages by Izawa et al. [30]. Thus, it is possible that further passages of between 8 and 43 passages of the Kenyan IBDV in the indigenous embryonated eggs could yield an attenuated virus that could be a candidate for a local vaccine.

The virus was successfully isolated from 44 out of the 67 AGPT-positive samples in this study. White leghorn chickens show very high susceptibility to IBDV and have been used by most investigators in IBDV experiments [34, 37]. Failure to isolate the virus from 23 of the 67 AGPT-positive field samples could be explained by the fact that AGPT detects viral antigen even in samples where the virus has been inactivated [12]. It means that even though the virus is hardy [38] samples intended for virus isolation experiments must be handled cautiously. In this study gross lesions and clinical signs of IBD were reported in all outbreaks from which 153 samples were collected, but viral antigen was only detected in 67 of the 153 samples submitted (out of which 44 samples yielded virus). It is possible that at the time of sampling the virus was not detectable by AGPT for that particular time of sampling in the course of the disease. The agar gel precipitation test (AGPT) detects viral antigen in the bursa of Fabricius in the early stages of the infection before the development of an antibody response [12].

Currently there is no concrete information on variations in disease susceptibility among indigenous chickens in Kenya; however, there is intention to determine variation among Kenyan indigenous chicken ecotypes and genotypes using innate and adaptive immunity [25]. Genetic divergence among chickens could result in individual variations in disease susceptibility and ability to transfer antibodies to progeny. In this study, however, the dams were tested and found to have no IBDV antibodies. In addition, the embryos were tested and confirmed negative for IBDV antibodies before use. Furthermore, the determinant factor for successful isolation of IBDV may be more of the antibody status and less of the host system, whether indigenous or exotic. Since the Kenyan indigenous populations are a heterogeneous population [39], genetic differences may affect the susceptibility of the chicken and the embryonated egg to IBDV infection. However, studies on IBD outbreaks across the country have shown that Kenyan indigenous chicken population is as susceptible to IBDV as the exotic ones [3].

The results of our study have shown, for the first time that Kenyan indigenous chicken embryos support the growth of IBDV and can be used to propagate the virus producing the typical lesions and with more passages could yield an attenuated vaccine.

## Figures and Tables

**Table 1:** Virus antigen detection by agar gel precipitation test in bursa of Fabricius collected from field outbreaks and experimental chicks.

Source of bursa of Fabricius	Agar gel precipitation test results			
	Number of positive	Number of negative	Total samples	Percentage of positive (%)
Chicks from suspected outbreaks (1st generation)	67	86	153	43.8
SPF chicks inoculated with field material (2nd generation)	44	23	67	65.7
Indigenous chicks inoculated with embryo propagated isolates (3rd generation)	44	0	44	100

**Table 2:** A summary of the lesions observed on inoculated embryos at different passages.

Lesion	Primary	Passage 1	Passage 2	Passage 3
Dwarfed embryo	112/132 (85%)	103/132 (78%)	106/132 (80%)	100/132 (76%)
Dead embryo	116/132 (88%)	124/132 (94%)	120/132 (91%)	88/132 (67%)
Congested embryo	45/132 (34%)	29/132 (22%)	20/132 (15%)	25/132 (19.0%)
Oedematous embryo	57/132 (43%)	77/132 (58%)	73/132 (55.0%)	75/132 (57%)
Haemorrhagic embryo	40/132 (30%)	53/132 (40%)	61/132 (46%)	63/132 (48%)
Enlarged mottled liver	26/132 (20%)	25/132 (19%)	18/132 (14%)	13/132 (10%)
Enlarged kidneys	4/132 (3%)	0%	0%	0%
Congested CAM	40/132 (30%)	36/132 (27%)	37/132 (28%)	32/132 (24%)
Haemorrhagic CAM	9/132 (7%)	15/132 (11%)	1/132 (1%)	13/132 (10%)
Oedematous CAM	71/132 (54%)	83/132 (63%)	87/132 (66%)	100/132 (76%)
